# Mitochondria-affecting small molecules ameliorate proteostasis defects associated with neurodegenerative diseases

**DOI:** 10.1038/s41598-021-97148-z

**Published:** 2021-09-06

**Authors:** Elissa Tjahjono, Jingqi Pei, Alexey V. Revtovich, Terri-Jeanne E. Liu, Alisha Swadi, Maria C. Hancu, Joe G. Tolar, Natalia V. Kirienko

**Affiliations:** Department of BioSciences, Rice University, 6100 Main St, MS140, Houston, TX 77005 USA

**Keywords:** Mitophagy, High-throughput screening, Phenotypic screening, Mitochondria, Alzheimer's disease

## Abstract

Macroautophagic recycling of dysfunctional mitochondria, known as mitophagy, is essential for mitochondrial homeostasis and cell viability. Accumulation of defective mitochondria and impaired mitophagy have been widely implicated in many neurodegenerative diseases, and loss-of-function mutations of PINK1 and Parkin, two key regulators of mitophagy, are amongst the most common causes of heritable parkinsonism. This has led to the hypothesis that pharmacological stimulation of mitophagy may be a feasible approach to combat neurodegeneration. Toward this end, we screened ~ 45,000 small molecules using a high-throughput, whole-organism, phenotypic screen that monitored accumulation of PINK-1 protein, a key event in mitophagic activation, in a *Caenorhabditis elegans* strain carrying a *Ppink-1*::PINK-1::GFP reporter. We obtained eight hits that increased mitochondrial fragmentation and autophagosome formation. Several of the compounds also reduced ATP production, oxygen consumption, mitochondrial mass, and/or mitochondrial membrane potential. Importantly, we found that treatment with two compounds, which we named PS83 and PS106 (more commonly known as sertraline) reduced neurodegenerative disease phenotypes, including delaying paralysis in a *C. elegans* β-amyloid aggregation model in a PINK-1-dependent manner. This report presents a promising step toward the identification of compounds that will stimulate mitochondrial turnover.

## Introduction

Although they are often simplistically characterized as the “powerhouse of the cell”, mitochondria have a wide range of cellular functions beyond that role, including amino acid metabolism, regulation of iron and calcium homeostasis, production of reactive oxygen species (ROS), stress surveillance, and control of apoptosis and other programmed cell death pathways^1–4^. Considering the number and variety of roles that they play, it is not surprising that mitochondrial maintenance is crucial for the health of cells and organisms.

Unfortunately, cells have few mechanisms to repair the damage mitochondria incur as they carry out their normal activities. When damage is limited, mitochondria fuse, allowing their content to be mixed and sorted, segregating the damaged material (by an unknown mechanism) into low-quality mitochondria targeted for destruction via macroautophagic degradation (hereafter referred to as mitophagy)^5^. The best-known pathway for triggering mitophagy is the PINK1/Parkin pathway, whose members have been linked to Parkinson’s disease^5–7^. Activation of this pathway begins with PTEN-induced kinase 1 (PINK1), a serine/threonine kinase that is constitutively expressed and targeted to mitochondria^5^. Upon arrival, healthy mitochondria import the kinase, leading to its prompt destruction by matrix-resident proteases. If mitochondria are damaged, PINK1 accumulates on the outer membrane instead, allowing it to cross-phosphorylate and activating its ability to phosphorylate other substrates, including the E3 ubiquitin ligase Parkin. Once phosphorylated, Parkin ubiquitinates its targets, including outer mitochondrial membrane proteins, which allows their recognition by the machinery that recruits the isolation membrane and begins the process of engulfing mitochondria into an autophagosome. Once the autophagosome has closed, it fuses with lysosomes and degradation of the contents begins.

Mitochondrial turnover is activated under certain physiological conditions, such as during the maturation of erythrocytes^8^, but evidence suggests that mitophagy participates in a broad variety of physiological functions, including in response to hypoxia and pathogen exposure^9–13^. Additionally, mitophagy is surprisingly important for the maintenance of normal function for neurological cells due to their high energy demands and reduced glycolytic capacity. For example, mutations in both PINK1 and Parkin are linked with early-onset Parkinsonism^6,14^. Mitochondrial dysfunction has been implicated in other neurodegenerative diseases, including Huntington’s disease, Alzheimer’s, and multiple sclerosis^7,15–18^, indicating that failed mitochondrial maintenance may be a common feature of these diseases.

Importantly, there is credible evidence that stimulating mitophagy can help mitigate some aspects of these disorders. For example, overexpression of PINK1 can restore mitophagy in a *Drosophila* model of Huntington’s disease and reduce disease symptoms^16^. However, the consensus opinion is that treating most of these disorders will be more effective using small molecule therapies, rather than genetic modifications^19^. Promising evidence in this area exists as well. In an elegant recent study, Fang and colleagues demonstrated that a number of molecules, including urolithin A, could stimulate mitophagy and reduce disease burden in *C. elegans*, human neuronal cells, and two murine Alzheimer’s disease models^20^. Additional efforts in this area have also been reviewed recently^19^.

To date, a diverse set of models have been developed to study neurodegenerative diseases in *C. elegans*^21–23^. This organism has several valuable traits, including a well-characterized nervous system, easy genetic manipulations, and highly-conserved neurological pathways^24,25^. *C. elegans* is amenable for low-cost and high-throughput compound screening due to its small size, short generation and lifespan, and the ability to simultaneously counter-screen for toxic compounds^24^. Finally, as *C. elegans* is transparent, intact worms can be used for high-content screens where more nuanced or complex phenotypes can be assessed.

In this study, we used a high-throughput, high-content phenotypic screen using *C. elegans* carrying GFP-tagged, full-length PINK-1/PINK1 (*Ppink-1*::PINK-1/PINK1::GFP) to identify a panel of small molecules that increase the level of PINK-1 protein (as monitored by the increased fluorescence of PINK-1::GFP fusion) and verified their effect on the activation of mitophagy and parameters of mitochondrial function. Two of the eight tested PS (**P**INK-1/**P**INK1 **S**tabilizer) molecules significantly reduced mitochondrial membrane potential (PS103 and PS106), three of the others reduced mitochondrial mass (PS34, PS127, and PS143), while another two affected both (PS30 and PS135, though the latter affected mitochondrial potential more significantly than mass). Neuroprotective properties of the selected PS compounds were tested in a transgenic *C. elegans* model of β-amyloid accumulation, where two of the compounds significantly delayed paralysis in a PINK-1-dependent manner. They were also able to reduce aggregate formation in a *C. elegans* polyglutamine-protein aggregation model. The compounds generally showed relatively low toxicity to human astroglial and prostate epithelial cells. This increases their promise as leads for future therapeutic development and corroborates the promise of affecting mitochondria in protein aggregation-triggered diseases.

## Results

### High-throughput screening of compound libraries identifies eight compounds that increase accumulation of PINK-1/PINK1 protein

**Figure 1:**
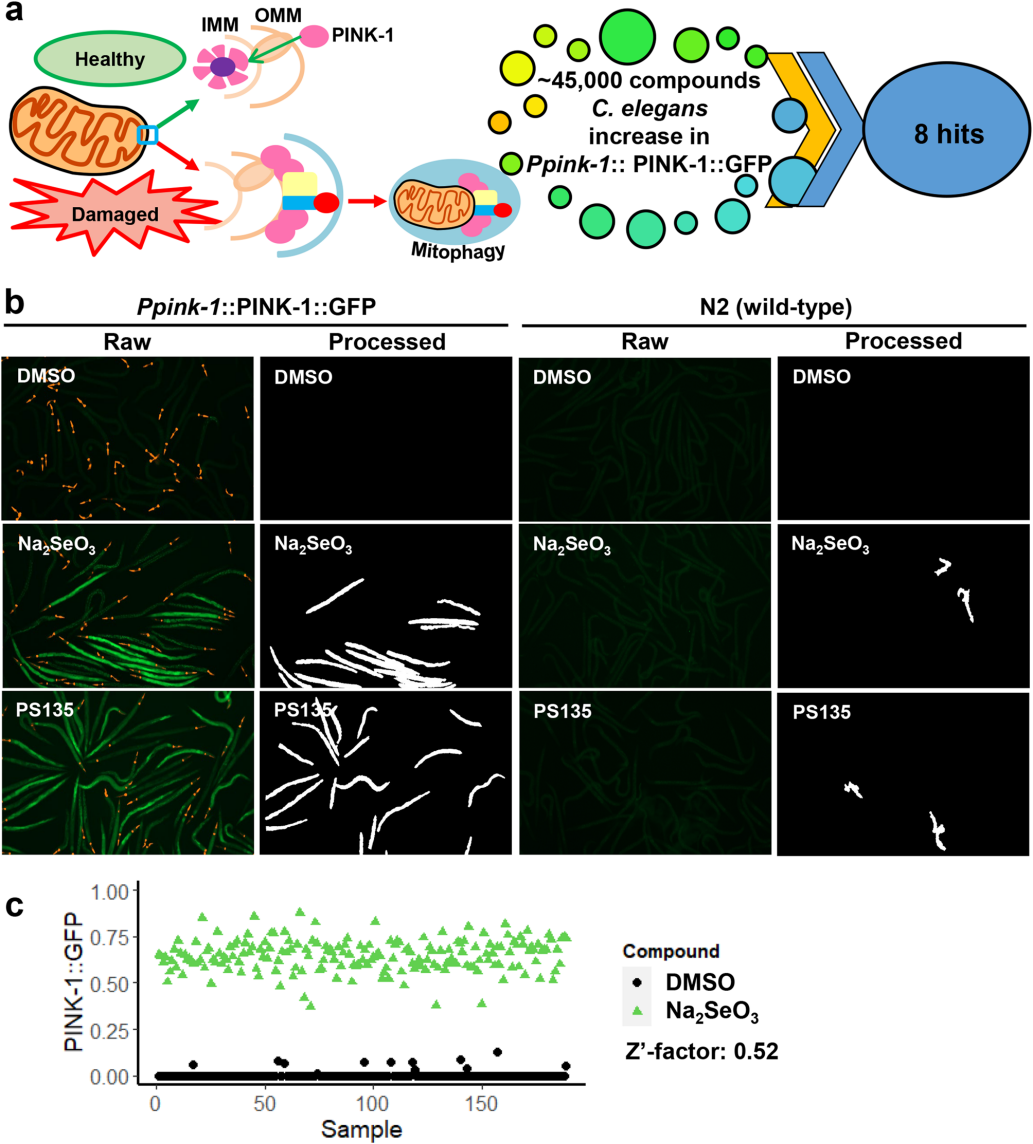
A high-throughput, high-content PINK-1/PINK1 stabilization screen yielded eight hit compounds. (**a**) The accumulation of PINK-1/PINK1 in the outer mitochondrial membrane of damaged mitochondria initiates mitophagy. 45,000 compounds were screened in *C. elegans* resulting in eight PINK-1/PINK1 stabilizers. (**b**) Fluorescent (raw) and cell profiler-processed (processed) images of *C. elegans* carrying *Ppink-1*::PINK-1/PINK1::GFP or N2 wild-type after 24 h of treatment with Na_2_SeO_3_, PS135, or DMSO control. Representative images are shown. (**c**) Quantification of GFP fluorescence of *C. elegans* carrying *Ppink-1*::PINK-1/PINK1::GFP after 72 h treatment with 7 mM Na_2_SeO_3_ or DMSO control. Three biological replicates for (**b**, **c**) were performed and analyzed.

As noted previously, preventing the degradation of PINK1 leads to mitophagic activation^26–28^ (Fig. 1a). To identify small molecules that promote PINK1 accumulation, we leveraged a *C. elegans* strain carrying a GFP-tagged, full-length PINK-1/PINK1 driven by its native promoter^9,29^. This reporter provides a simple method to track accumulation of non-degraded PINK-1/PINK1 by visualizing GFP. To develop a high-throughput, high-content phenotypic screen in *C. elegans*, we optimized parameters by identifying efficient conditions for the activation of mitophagy by sodium selenite (Na_2_SeO_3_), which triggers the production of mitochondrial superoxide^30,31^ (Fig. 1b).

Worms were exposed to the negative control or to sodium selenite for 24 h and then GFP was visualized and quantified using an automated pipeline (to eliminate researcher bias) by using Cell Profiler software^32,33^ (Raw vs. Processed, respectively, Fig. 1b). We determined the Z′-factor of the assay to be 0.52 (Fig. 1c). Z′-factors range from − ∞ to 1, with the score indicating the degree of separation of the means and variability of the positive and negative controls, which indicates the ability of the assay to discriminate between signal and noise. A Z′-factor > 0.5 indicates an ability to detect even moderately weak hits.

Using this assay, approximately 45,000 wells from various small molecule diversity and targeted libraries were tested for the ability to increase PINK-1/PINK1::GFP-signal. Primary hits were counter-screened in wild-type worms (which do not express GFP) to eliminate the possibility that the compounds themselves were fluorescent in worms. The eight compounds identified in this way were given the following **PS** (for **P**INK-1/PINK1 **S**tabilizer) designations: PS30, PS34, PS83, PS103 (triclosan), PS106 (sertraline), PS127, PS135, and PS143 (Fig. S1, Table S1). The final hit rate was 0.018%, which is somewhat lower than is commonly found from high-content screening in *C. elegans*^34,35^, demonstrating selectivity and specificity of the assay. Compound similarity analysis yielded Tanimoto coefficients^36^ that suggested substantial structural differences amongst the eight compounds (Table S2). This outcome was not surprising, as the hits primarily came from diversity libraries that were intended to explore broad chemical space.

### PS compounds trigger mitochondrial fragmentation and autophagosome formation

**Figure 2:**
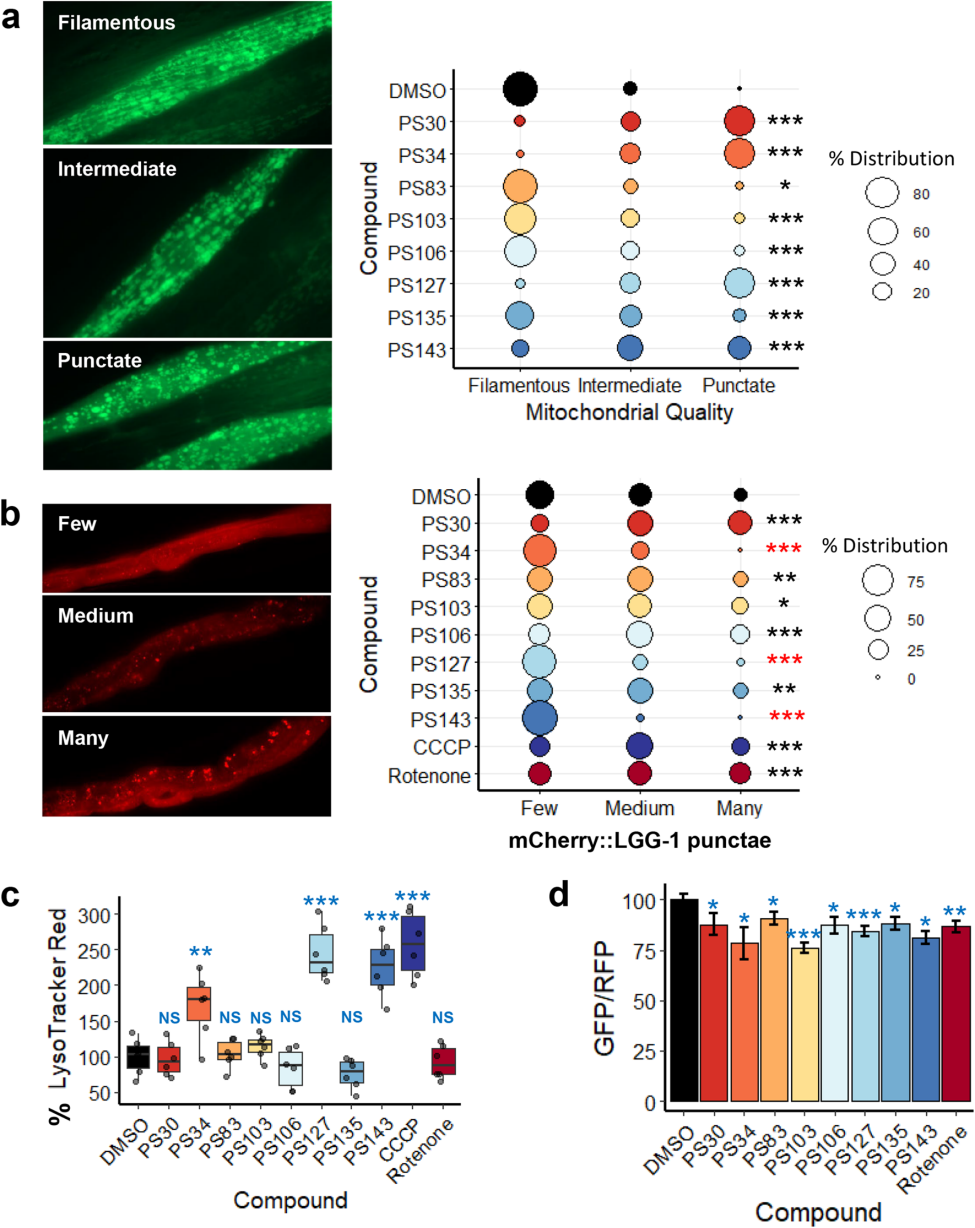
PS compounds induced mitochondrial fragmentation and mitophagy. (**a**, **b**) Fluorescent images and quantification of fluorescence of *C. elegans* carrying (**a**) *Pmyo-3*::GFP^mt^ or (**b**) mCherry::LGG-1/LC3 upon 15 h of treatment with PS compounds. (**c**) Quantification of LysoTracker Red fluorescence upon 15 h of treatment with DMSO, PS compounds, CCCP, or rotenone. (**d**) GFP/RFP ratio of Rosella^mt^ upon 15–34 h of treatment with PS compounds. For (**a**, **b**), percent distribution for each category was calculated and plotted, Chi-square statistic tests were performed, and representative images are shown. Three biological replicates with ~ 30 worms/replicate were analyzed. For (**c**), at least four biological replicates with ~ 400 worms/replicate were analyzed. For (**d**), at least three biological replicates with ~ 20 worms/replicate were analyzed. Representative images for (**d**) were shown in Fig. S2. For (**c**, **d**), *p*-values were determined from one-way ANOVA, followed by Dunnett’s test. All fold changes were normalized to DMSO control (at 100%), thus increased fluorescence (as compared to DMSO) will cause quantification values to be over 100%. NS not significant, *p < 0.05, **p < 0.01, ***p < 0.001. In (**b**), black star indicates significance of increase while red indicates significance of decrease of mCherry::LGG-1 punctae formation.

As stabilization or accumulation of PINK-1/PINK1::GFP fusion can be achieved via multiple direct and indirect mechanisms, we proceeded to further characterize these molecules. As the first step of characterizing the activity of the PS compounds on mitochondria, their impact on mitochondrial morphology was tested using a strain of *C. elegans* that expresses a mitochondrially-targeted GFP in body wall muscles (*Pmyo-3*::GFP^mt^). Under normal circumstances, these mitochondria take on a long, branched tubular network architecture that lies along muscle fibers. Worms treated with DMSO mostly showed this state (> 85%, Fig. 2a).

Qualitative analysis of the mitochondrial network structure showed that treatment with the PS compounds significantly increased the tendency towards intermediate (partially fragmented) or punctate (strongly fragmented) mitochondrial networks. Four of the compounds, PS30, PS34, PS127, and PS143, induced substantial fragmentation, as shown by the dissolution of the tubular network into discrete punctae. PS83, PS103, PS106, and PS135 induced a milder disruption of the mitochondrial network, although each was statistically significant. This is consistent with observations that disruption is a necessary first step for the autophagic degradation of large mitochondrial networks^10,37^.

To confirm that compound exposure triggers autophagosome formation, we exposed a worm strain expressing mCherry::LGG-1 to the PS compounds. Under normal conditions, LGG-1, like its mammalian ortholog MAP1LC3, exhibits a diffuse cytoplasmic localization^38^. During the formation of the isolation membrane (which later becomes the autophagosome), LGG-1/MAP1LC3 is crosslinked to the lipids that will comprise this membrane, converting the localization from a diffuse pattern into bright punctae^38,39^. Punctae were qualitatively assessed as few, medium, or many, with representative images shown (Fig. 2b). Compared to the DMSO control, several of the PS compounds increased punctae localization of mCherry::LGG-1/LC3, including two strong hits (PS30 and PS106) and three weak hits (PS83, PS103, and PS135), indicating increased autophagosomal formation. Intriguingly, three compounds (PS34, PS127, and PS143) reduced the production of autophagosomes, an unexpected outcome from a treatment that stimulates the accumulation of PINK-1/PINK1. Two mitochondrial disruptors, rotenone (which blocks Complex I of the electron transport chain (ETC)) and carbonyl cyanide *m-*chlorophenyl hydrazine (CCCP, a proton uncoupler that dissipates the electrochemical gradient on the mitochondrial membrane) (Fig. 2b), were used to validate the assay.

After formation of the autophagosome is completed, the next step in mitophagy is for the autophagosome to fuse with lysosomes to form autophagolysosomes^40^. To observe this process, worms were treated with PS compounds, CCCP, rotenone, or vehicle control for 15 h. Afterward, worms were stained with LysoTracker Red DND-99, a dye that specifically labels acidic cellular compartments. This dye is routinely used to label lysosomes and autophagolysosomes^41,42^. Uptake was quantitatively measured using flow vermimetry^43^. Increased fluorescence was seen after treatment with PS34, PS127, PS143, and CCCP, but not with the rest of the PS compounds (Fig. 2c).

In contrast, treatment with PS30, PS103, PS106, or PS135 fragmented mitochondria but did not result in substantial formation of acidified organelles, at least by 15 h. A similar outcome was also seen after treatment with rotenone, which triggered the early stages of mitophagy (i.e., PINK-1/PINK1 stabilization, formation of the isolation membrane and autophagosome), but did not proceed to autophagosomal acidification. This is consistent with reports elsewhere regarding the accumulation of autophagosomes and decreased autophagic completion after rotenone exposure^44,45^. The ability of rotenone to prevent autophagy has been attributed to depletion of ATP, which limits the lysosomal vacuolar ATPase from consuming ATP to acidify the autophagolysosome^44^.

As an independent method of assessing acidification of the autophagolysosome, we utilized a worm strain that expresses the Rosella reporter that is trafficked to mitochondria by fusing the reporter to the mitochondrial targeting sequence from the mitochondrial translocase TOMM-20^46^. The Rosella reporter itself is a fusion protein of GFP (which is sensitive to an acidic pH, and loses fluorescence at low pH) and RFP (which is insensitive to pH and remains fluorescent at low pH). Consequently, Rosella^mt^ in healthy, cytoplasmic mitochondria will fluoresce both green and red, and will appear yellow. In contrast, Rosella^mt^ in mitochondria that are being degraded in autophagolysosomes, where the pH has dropped to ~ 4, only fluoresce red. After 15 to 34 h of treatment (the variation in treatment length resulted from differences in compound toxicity), all eight PS compounds and rotenone reduced the ratio of GFP/RFP of the reporter, albeit none to a strong degree (Fig. 2d, Fig. S2). The difference in outcomes of mitochondrial fragmentation, LGG-1/MAP1LC3 localization, and formation of acidified autophagolysosomes assays suggested that the compounds are acting via several distinct mechanisms (see Table 1 for the summary of these and other phenotypes).

**Table 1:** Summary of PS compounds’ effects on various mitochondrial parameters and other cellular pathways.

	PS30	PS34	PS83	PS103	PS106	PS127	PS135	PS143
Mt fragmentaton	**High**	**High**	*Low*	Mid	Mid	**High**	Mid	**High**
Autophagosome formation	**High**	*Less*	Mid	Mid	Mid	*Less*	Mid	*Less*
Autophagolysosome formation	NS	**High**	NS	NS	NS	**High**	NS	**High**
ATP depletion	**High**	**High**	NS	NS	NS	**High**	**High**	**High**
O_2_ consumption rate reduction	NS	**High**	NS	NS	NS	**High**	NS	NS
Mt mass reduction	NS	**High**	NS	NS	NS	**High**	NS	NS
Mt ΔΨ reduction	**High**	NS	NS	**High**	Mid	NS	**High**	NS
ROS formation	NS	NS	NS	NS	NS	NS	NS	NS
GST-4 (SKN-1/Nrf2) induction	NS	Mid	Mid	NS	NS	Mid	NS	Mid
DAF-16/FOXO nuclear loc	NS	**High**	**High**	NS	NS	**High**	NS	**High**
IRG-5 (immune response)	**High**	NS	NS	NS	NS	NS	**High**	**High**

*NS* not significant.

### PS compounds show different effects on multiple mitochondrial parameters

**Figure 3:**
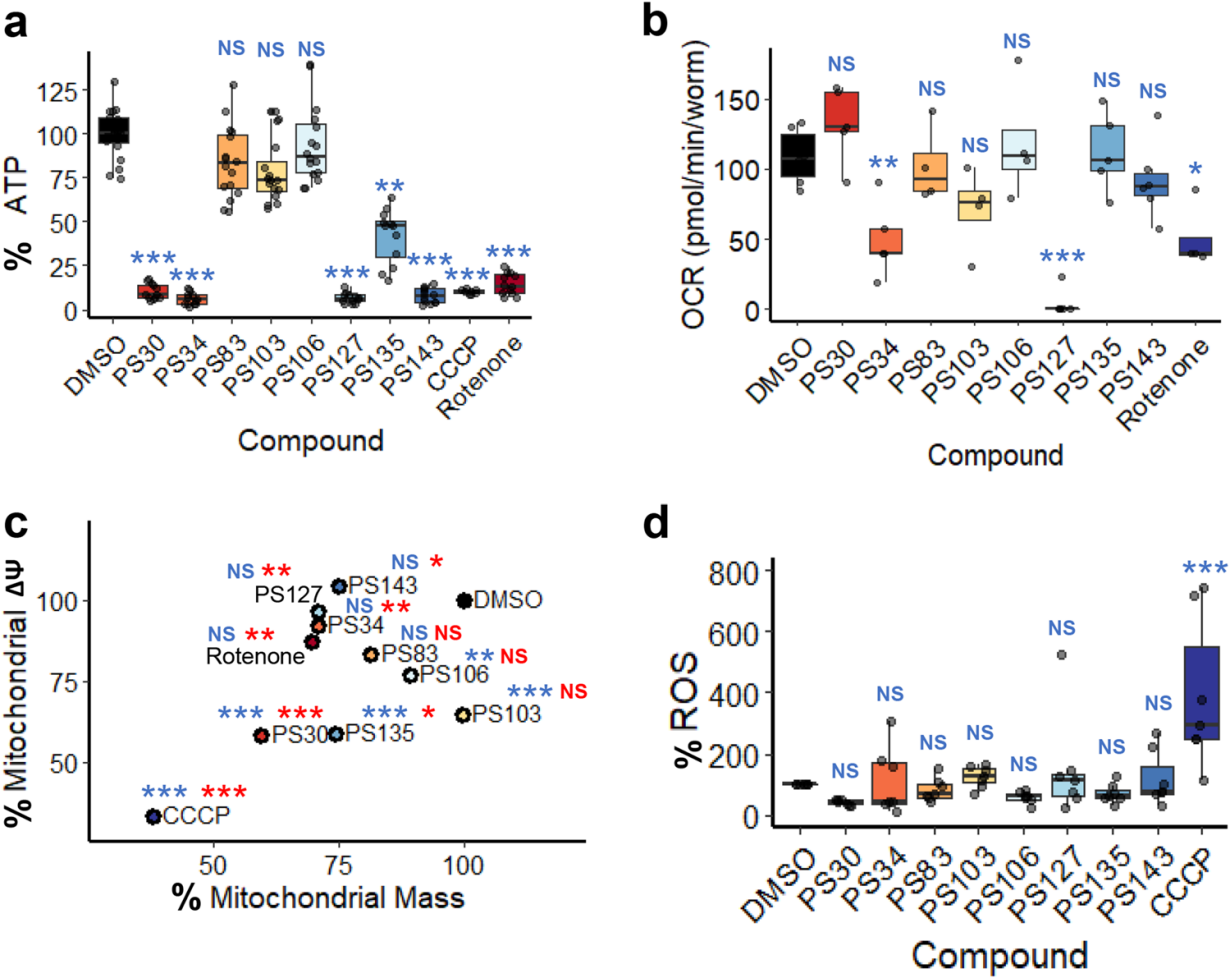
PS compounds differently affected multiple mitochondrial parameters. (**a**) Quantification of luminescence (normalized to GFP) of *C. elegans* carrying *Psur-5*::luciferase::GFP upon 19 h of treatment with DMSO, PS compounds, CCCP, or rotenone. (**b**) Oxygen consumption rate (pmol/min/worm) measurement of wild-type worms upon 8 h of treatment with PS compounds or rotenone. (**c**) Point plot of MitoTracker Green (mitochondrial mass, *x*-axis) and MitoTracker Red (mitochondrial membrane potential, *y*-axis) fluorescence of wild-type worms upon 15 h of treatment with PS compounds or CCCP. (**d**) Quantification of DHE fluorescence upon 15 h of treatment with PS compounds or CCCP. At least three biological replicates with (**a**, **c**, **d**) ~ 400 worms/replicate or (**b**) 6000 worms/replicate were analyzed. *p*-values were determined from one-way ANOVA, followed by Dunnett’s test. For (**a**, **c**, **d**), fold-changes were normalized to DMSO control (at 100%). NS not significant, *p < 0.05, **p < 0.01, ***p < 0.001. In (**c**), blue color indicates significance of change in mitochondrial membrane potential; red indicates significance of change in mitochondrial mass.

To test the impact of compound treatment on mitochondrial health and function, first we tested ATP levels in compound-treated worms carrying a ubiquitously expressed firefly luciferase^47,48^. Worms were treated with each of the eight molecules. Compounds that greatly induced mitochondrial fragmentation (PS30, PS34, PS127, and PS143, see Fig. 2a) also caused significant drop in ATP production (Fig. 3a). Unexpectedly, there was no apparent correlation between ATP depletion and failure to acidify autophagolysosomes. To investigate whether the failure of ATP production was due to the inhibition of the ETC, we monitored the last step of the electron transport by measuring oxygen consumption rate (OCR)^49^. Only PS34, PS127, or rotenone significantly lowered oxygen consumption rate (with PS127 completely abolishing respiration) (Fig. 3b).

Next mitochondria were stained with MitoTracker Red, which accumulates in mitochondria proportionally to their membrane potential, and with MitoTracker Green, a dye that covalently binds to mitochondrial proteins (free thiol groups of cysteine residues) and is comparatively insensitive to mitochondrial membrane potential. The difference in staining allows for a comparison of mitochondrial mass and membrane potential in worms treated with either the PS compounds or a solvent control (Fig. 3c). However, it is difficult to unambiguously interpret MitoTracker Green data, as CCCP, which dissipates mitochondrial membrane potential and induces mitophagy^50^, reduced both mitochondrial mass and mitochondrial membrane potential to a similar extent.

Significant reduction of mitochondrial membrane potential was observed in worms treated with PS30, PS103, PS106, and PS135 (Fig. 3c). For two of the compounds (PS103 and PS106), this reduction did not appear to be accompanied by a corresponding decrease in mitochondrial mass, which may be indicative of weak uncoupling activity. Meanwhile, treatment with PS30 and PS135 substantially reduced mitochondrial membrane potential and mass, indicating that these two compounds may also have uncoupling activity. These data suggest that for at least four compounds, the loss of mitochondrial membrane potential may contribute to mitophagic activation. In contrast, treatment with PS34, PS127, or PS143 reduced apparent mitochondrial mass, but not mitochondrial membrane potential, which may indicate the presence of fewer mitochondria, but with increased membrane potential. We previously observed similar changes when worms’ diet was supplemented with vitamin B12, which improved mitochondrial health^51^. PS83 did not significantly affect mitochondrial membrane potential or mass (Fig. 3c). Combined, these results suggest that the loss of ATP content in PS30 and PS135 was not due to failure of the mitochondrial ETC, as in the case for PS34, PS127, and PS143. Instead, PS30, PS103, PS135, and, to a lesser extent, PS106 may have mild uncoupling activity.

Another common signal for the induction of mitophagy is the accumulation of ROS^52,53^. To test whether the PS compounds induced ROS production, worms were treated with compounds for 15 h, and then were stained with dihydroethidium, a non-fluorescent, redox-sensitive dye that is converted to fluorescent 2-hydroxyethidium by reaction with superoxide^54^. Surprisingly, none of the compounds appeared to significantly increase ROS production (Fig. 3d). CCCP was used as a positive control to validate the assay (Fig. 3d).

Considering that the PS compounds were initially found based on increased intensity of PINK-1/PINK1::GFP signals, we examined whether the mechanisms of action of these compounds were dependent on the presence of PINK-1/PINK1. For two of the compounds (PS30 and PS135), knockdown of *pink-1* significantly reduced mitochondrial fragmentation (Fig. S3). To assess the impact of *pink-1(RNAi)* on mitochondrial mass and membrane potential, we selected compounds that decreased these parameters in wild-type worms (PS127 for mitochondrial mass, and PS106 and PS135 for membrane potential). We observed increased mitochondrial mass on *pink-1(RNAi)* worms upon exposure to PS127 (the compound that strongly reduced mitochondrial mass but not mitochondrial membrane potential) (Fig. S4a). However, we did not see any difference in mitochondrial membrane potential between *pink-1(RNAi)* and empty vector (EV) worms when treated with compounds that reduced mitochondrial membrane potential (PS106 and PS135) (Fig. S4b), indicating that membrane depolarization acted upstream of mitophagic activation via PINK-1/PINK1.

### Four PS compounds trigger DAF-16/FOXO nuclear localization and SKN-1/Nrf2 pathway activation

**Figure 4:**
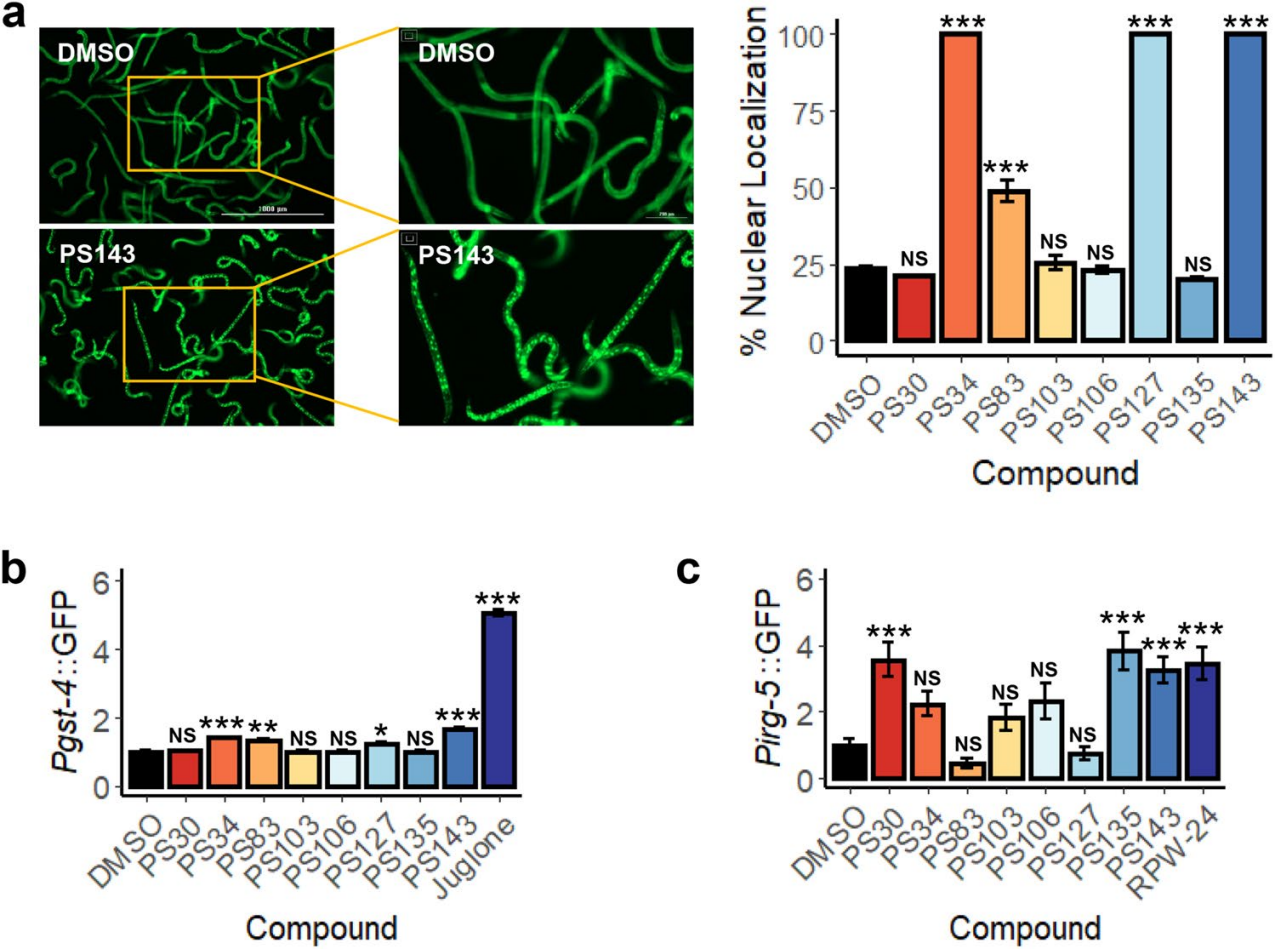
Four of the eight PS compounds activated DAF-16/FOXO and SKN-1/Nrf2 pathways. (**a**) Fluorescent images and quantification of nuclear localization (%) of *C. elegans* carrying *Pdaf-16*::DAF-16a/b::GFP upon 4.5 h of treatment with PS compounds. (**b**, **c**) Quantification of fluorescence of *C. elegans* carrying (**b**) *Pgst-4*::GFP or (**c**) *Pirg-5*::GFP upon 8 h of treatment with PS compounds. Representative images are shown in (**a**). Three biological replicates with ~ 400 worms/replicate were analyzed. Error bars represent SEM. *p*-values were determined from one-way ANOVA, followed by Dunnett’s test. All fold changes in (**b**, **c**) were normalized to DMSO control. NS not significant, *p < 0.05, **p < 0.01, ***p < 0.001.

Autophagic activation integrates a large number of signals, including stress and nutrient status. For this reason, we tested whether two master stress response pathways, the DAF-16/FOXO pathway and the SKN-1/Nrf pathway, are activated by exposure to the hit compounds. For DAF-16, a worm strain carrying a *Pdaf-16*::DAF-16a/b::GFP translational reporter^55^ was treated with each of the compounds. Four compounds (PS34, PS83, PS127, and PS143) triggered DAF-16 translocation into the nucleus, indicating that it has been activated (Fig. 4a). The same four compounds also activated the conserved SKN-1/Nrf pathway, as shown by a transcriptional reporter (*Pgst-4*::GFP^56^) commonly used to assess SKN-1 activation, albeit to a lower level of activation than DAF-16 (Fig. 4b). Intriguingly, SKN-1 has been linked to mitophagy directly; when mitochondrial turnover pathways are compromised, SKN-1 is activated^46^. The consequences of this activation include increased mitochondrial biogenesis, which may be an attempt of the organism to resolve the mitochondrial stress by ‘diluting’ the damaged components.

Mitochondrial dysfunction that is sufficient to trigger mitophagy has also been shown to activate innate immune pathways^57–59^. To test whether immune activation occurs after treatment with these compounds, a worm strain carrying *Pirg-5*::GFP, a transcriptional reporter for the PMK-1 innate immune pathway^60^, was exposed to the PS compounds or to RPW-24, a positive control. Only three of the compounds, PS30, PS135, and PS143, induced PMK-1 pathway activity (Fig. 4c).

### Two compounds, PS83 and PS106, can improve proteostasis

Defects in mitochondrial clearance have long been linked to diminished physiological function and neurodegenerative diseases (as reviewed in^61^). On this basis, it is reasonable to hypothesize that the impacts of the compounds on mitochondria may stimulate protective effects in *C. elegans* models of neurodegenerative diseases.

**Figure 5:**
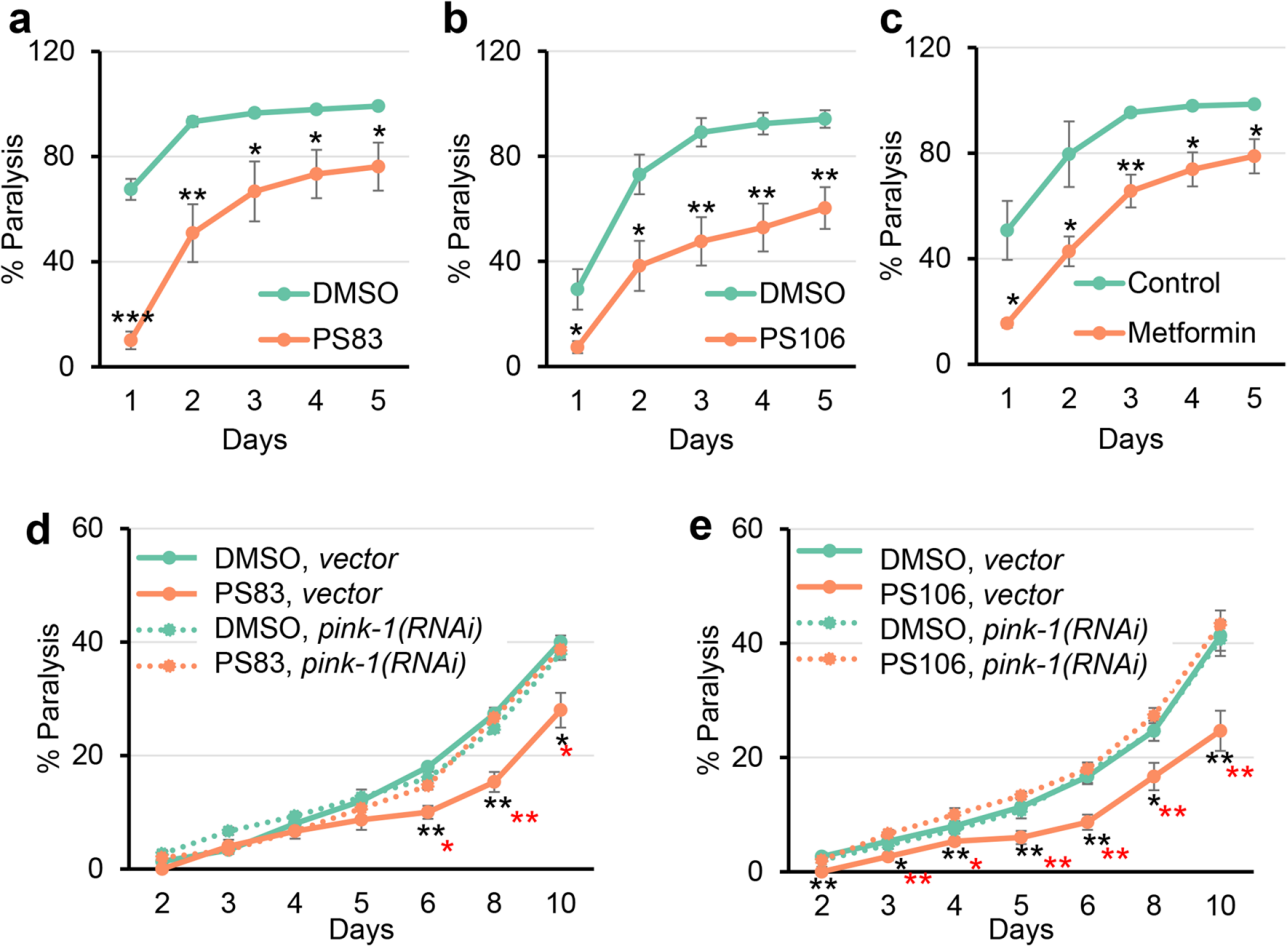
PS83 and PS106 reduced the rate of paralysis in *C. elegans* strain expressing human beta-amyloid. (**a**–**e**) Rate of paralysis curve of *C. elegans* Alzheimer’s model (GMC101) expressing full-length human beta-amyloid upon treatment with (**a**, **d**) 5 µM PS83, (**b**, **e**) 25 µM PS106, or (**c**) 100 mM metformin control. In (**d**, **e**), worms were reared on *E. coli* expressing *cdc-25.1(RNAi)/vector(RNAi)* or *cdc-25.1(RNAi)/pink-1(RNAi)*. At least three biological replicates with ~ 180 worms/replicate were analyzed. *p*-values were determined from Student’s *t*-test. *p < 0.05, **p < 0.01, ***p < 0.001. Black stars indicate significance compared to DMSO control. Red stars in (**d**, **e**) indicate statistical significance between PS compound on *vector* versus *pink-1(RNAi)*-reared animals.

A *C. elegans* model of β-amyloid accumulation was used to test this prediction. This strain, GMC101, produces full-length human β-amyloid in body wall muscles^62^. Once the peptide has been expressed, shifting the strain to a higher temperature leads to β-amyloid aggregation and paralysis. Each hit compound was tested at 2–4 different concentrations (Fig. S5). Two compounds, PS83 and PS106, substantially reduced paralysis at 5 µM and 25 µM, respectively, and were comparable to the positive control metformin (Fig. 5a–c and Fig. S5). Another compound, PS103, provided a more modest, but still significant, decrease in paralysis at 25 µM (Fig. S5).

Formally, it is possible that PS83- and PS106-mediated rescue was independent of the accumulation of PINK-1 that was caused by the compounds. To test this, RNAi was used to knock down *pink-1* expression in the β-amyloid-expressing strain prior to its exposure to the PS compounds. Consistent with our interpretation, *pink-1(RNAi)* completely removed the ability of PS83 or PS106 to delay paralysis, and made worms treated with PS83 or PS106 indistinguishable from vehicle controls (Fig. 5d, e). This is in agreement with findings presented in Du et al., where increased PINK-1 expression in β-amyloid-enriched brains improved mitochondrial synaptic function and reduced amyloid pathology in a murine Alzheimer’s disease model^63^. As Du et al. observed, the blockade of autophagosome LC3-II activation failed to rescue amyloid pathology through PINK1 overexpression^63^. These data suggest that PINK1-dependent mitophagic signaling may more generally suppress amyloid pathology.

To test whether the compounds had a broader effect on proteostatic disruptions associated with neurodegenerative diseases, we obtained a worm strain that expresses a YFP-tagged protein with an engineered polyglutamine (polyQ) repeat of 82 consecutive glutamine residues (Q82::YFP)^64^. PolyQ repeats are causative factors for at least ten different neurodegenerative diseases, with the best-known being Huntington’s chorea^65^. Expression of the chimeric Q82::YFP product under the control of a tissue-specific promoter (e.g., *unc-54* or *vha-6*) has been shown to result in the aggregation of the protein in the target tissue^64^.

**Figure 6:**
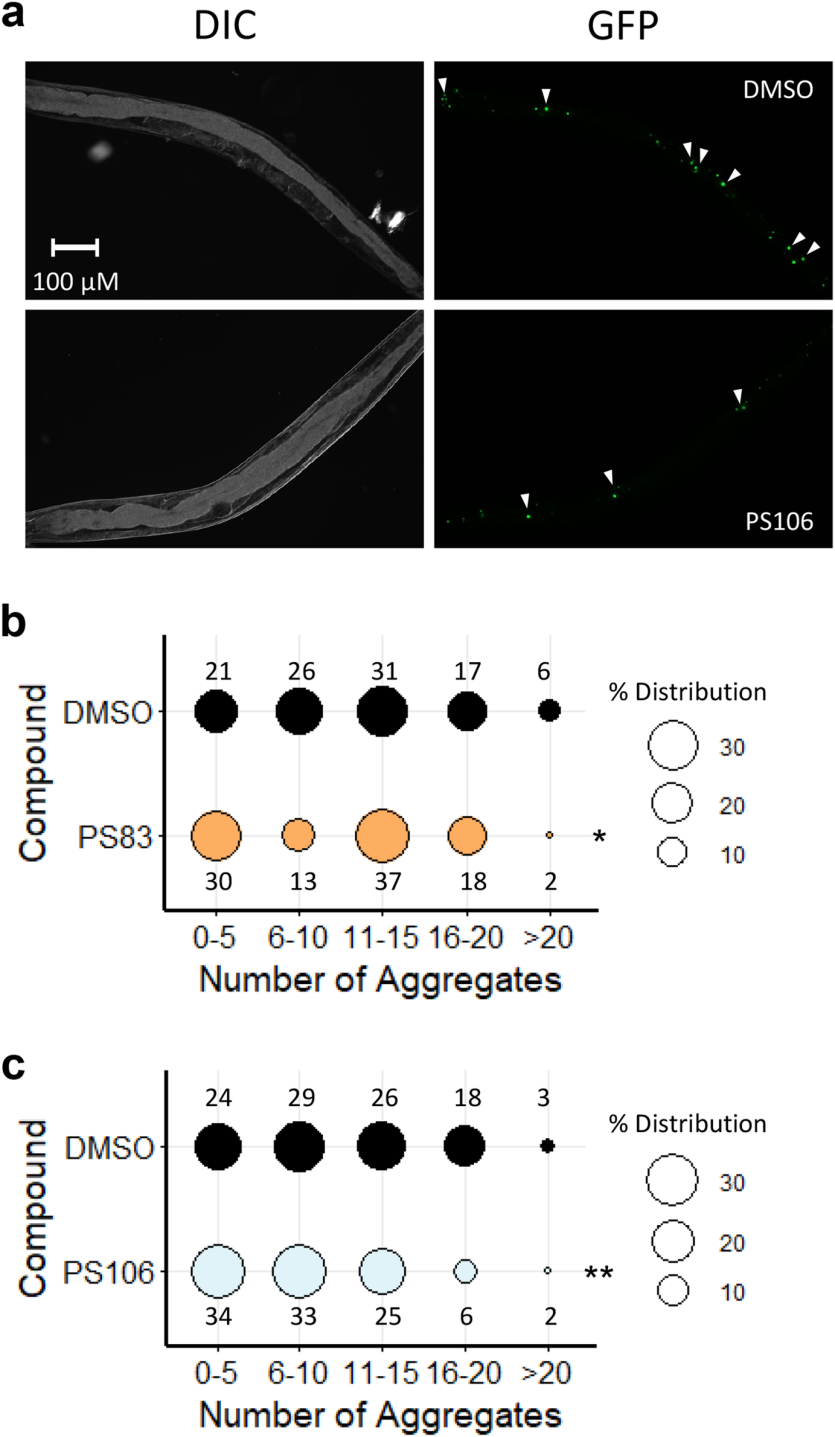
PS106 reduced aggregate formation in *C. elegans* strain expressing polyglutamine. (**a**) Representative DIC and GFP images of *C. elegans* strain expressing polyglutamine (Q82) (GF66) upon 36 h of treatment with DMSO or 25 µM PS106. (**b**, **c**) Percent distribution of the number of polyglutamine aggregates upon treatment with (**b**) 5 µM PS83 or (**c**) 25 µM PS106. Three biological replicates with ~ 30 worms/replicate were scored and analyzed. *p*-values were determined from Chi-square test. *NS* not significant, *p < 0.05, **p < 0.01.

Young adult worms expressing the Q82::YFP construct under the intestinal promoter *vha-6* (Fig. 6a) were treated with PS83 (5 µM) or PS106 (25 µM) for 36 h, and then aggregates were manually counted under low magnification (Fig. 6). We found that PS83 showed a slight but significant difference from vehicle alone (Fig. 6b), while PS106 treatment more significantly reduced the number of aggregates (Fig. 6c). Previously, the Morimoto lab implicated DAF-16/FOXO activation in limiting Q82::YFP aggregation and paralysis when aggregates form in body wall muscles^64^. Since PS106 did not induce DAF-16 nuclear localization, the precise regulatory pathway induced by PS106 and its role in providing neuroprotection will need to be further elucidated.

Interestingly, a recent report linked proteostatic defects, such as accumulation of β–amyloid or glutamine repeat proteins, to increased mitochondrial genome heteroplasmy, especially in neurons^66^. If PS83 or PS106 can alleviate these proteostatic defects and promote mitochondrial turnover, they may reduce this heteroplasmy, which is associated with decreased mitochondrial function, reduced cell viability, and chronic, degenerative disease.

### PS compound exposure shows limited toxicity

**Figure 7:**
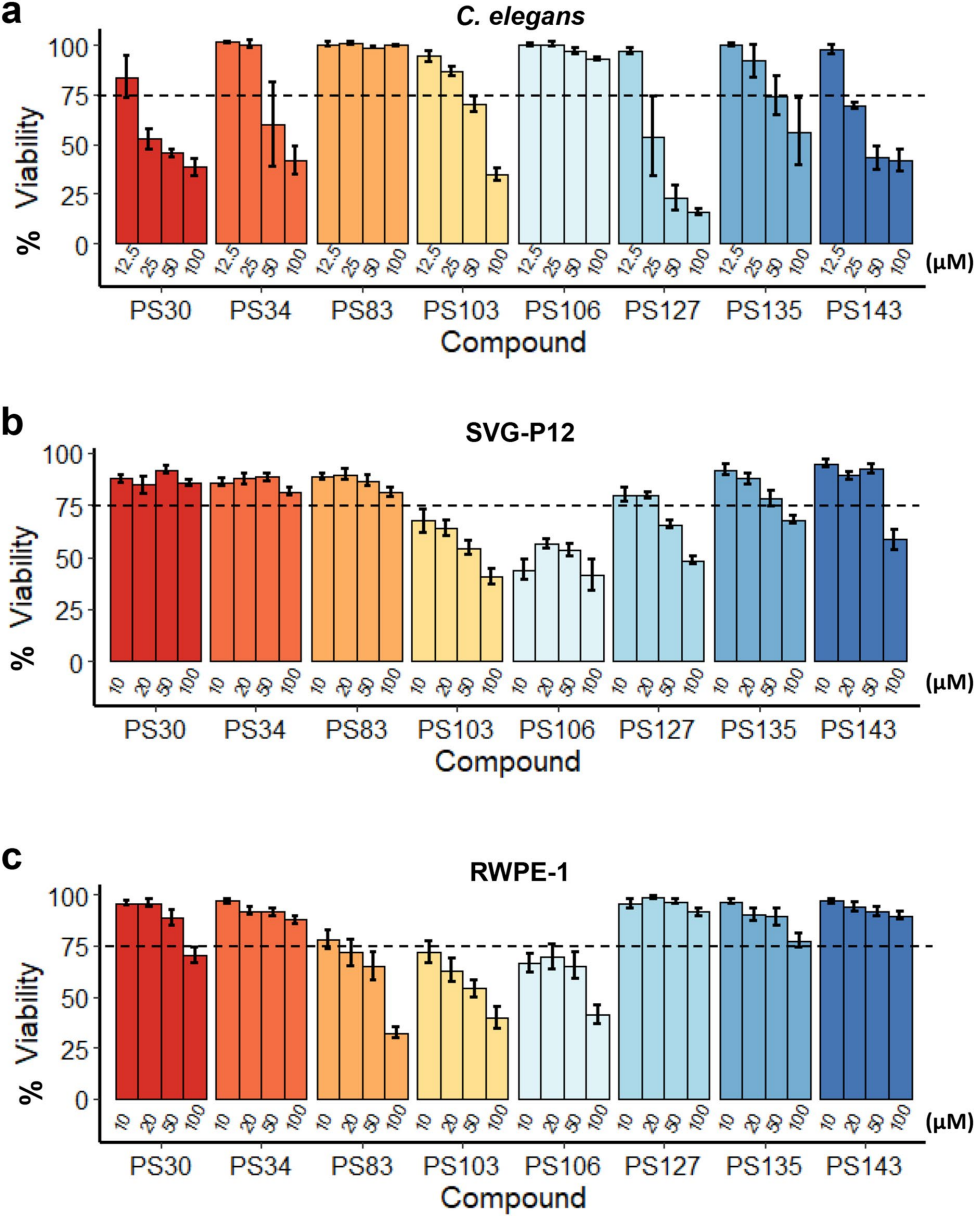
Most PS compounds have low toxicity in *C. elegans* and mammalian cells. Viability of (**a**) *C. elegans*, (**b**) human astroglial (SVG-P12) cell line, or (**c**) human prostate epithelial cell line (RWPE-1) upon 3 days of exposure to PS compounds with concentration gradient as indicated on the graphs. Dotted lines mark 75% of viability. At least three biological replicates were analyzed. Error bars represent SEM. All fold changes were normalized to DMSO control.

While activation of mitophagy may provide a route to mitigate some aspects of neurodegenerative disease, overactivation of mitophagy may lead to excess mitochondrial loss, bioenergetic deficits, and cellular death^67^. To test whether prolonged exposure to PS compounds causes death, survival of *C. elegans* and human cells was measured. For *C. elegans*, worms were exposed to the eight PS compounds at four different concentrations for 72 h and then were incubated with Sytox Orange, a cell-impermeant dye that stains DNA in dead worms. Treatment with most compounds showed > 75% survival (as normalized to the DMSO control) at concentrations of 25 µM or less (Fig. 7a). In all cases, worms survived the lowest tested dosage; in all but one (i.e., PS83), the highest dose caused at least partial death (Fig. 7a). This suggests that dosage optimization would be necessary to see protective effects. Importantly, the two compounds that reduced paralysis rate in the *C. elegans* Alzheimer’s model (PS83 and PS106) did not impair survival at tested concentrations of up to 100 µM (Fig. 7a).

Cytotoxicity of chronic, 72 h exposure of PS compounds at similar concentrations was also measured in two human cell lines, SVG-P12 (an astroglial cell line) and RWPE-1 (prostate epithelial cells) (Fig. 7b, c). Differential Hoechst/propidium iodide staining, which we recently showed is comparatively insensitive to mitochondrial dysfunction^68^, was used to assess cell death. Exposure to either PS103 or PS106 at concentrations higher than 10 µM showed substantial toxicity in both cell lines. Surprisingly, four of the compounds (PS34, PS127, PS135, and PS143) showed greater toxicity in *C. elegans* than in human cells.

## Discussion

Stimulation of mitophagy has proven to be a promising therapeutic target for neurodegenerative diseases^69^ and may be beneficial for healthy aging. Using a high-throughput, high-content phenotypic screen, we obtained and characterized eight compounds that lead to increased accumulation of PINK-1/PINK1. We are careful to note that we have not yet identified molecular mechanism(s) for these eight compounds; it remains possible that their effect is indirect and that the prevention of PINK-1 degradation occurs as a consequence of mitochondrial damage. However, given cells’ ability to fuse, sort, and separate mitochondria into healthy and diseased organelles, it is not clear that such a mechanism would be an insurmountable obstacle. Previous research in our lab has shown that healthy cells can withstand a certain amount of mitochondrial damage without apparent ill effect, and that this form of treatment can be beneficial^70^.

Interestingly, two of the compounds, PS103 and PS106, had previously been associated with alterations in mitochondrial function. Both also induced considerable mitochondrial fragmentation and loss of membrane potential in worms (Table 1). PS103, commonly known as triclosan or irgasan, has been linked to a variety of mitochondrial dysfunctions, including uncoupling of the mitochondrial membrane potential by reversible protonation of the compound’s phenoxy group^71–73^ and inhibition of Complex II of the ETC^74^. Triclosan has also been associated with increased mitochondrial ROS, reduced mitochondrial mass, and disruptions in mitochondrial morphology^75^. The ability of triclosan to cause several types of mitochondrial damage, apparently with different proximal factors^74^, somewhat reduces its value as a therapeutic agent.

Despite its common appearance in a wide variety of consumer products, questions about the safety of triclosan remain, even if used externally. For example, in addition to its role in mitochondrial disruption, triclosan also has the potential to disrupt endocrine function, affect immunity, disrupt calcium and zinc homeostasis, and alter lipid metabolism^76^. Triclosan also has a strong potential for bioaccumulation^77^, which is undesirable in a maintenance medicine. Given these caveats, the potential for triclosan to be developed into a treatment for NDD seems small.

The potential for PS106, more commonly known as sertraline, is substantially greater. Sertraline is thought to bind to the serotonin transporter (SERT) in the presynaptic neuron, preventing reabsorption of serotonin and prolonging synaptic signaling. Sertraline is a well-known compound with carefully studied pharmacological effects and is one of the most commonly prescribed psychiatric medications in the US. Our data contribute to an ongoing discussion about the potential for sertraline as a treatment for one or more NDDs.

Probably the earliest hint that sertraline may have some unexpected effect on mitochondria came in a report attempting to identify ‘hidden’ drug targets, where the authors determined that sertraline had several characteristics similar to the well-known mitochondrial toxin rotenone^78^. Not long after, Kumar and colleagues demonstrated that sertraline treatment could ameliorate damage caused by the mitotoxic agent 3-nitropropionic acid^79^. Recently, it was demonstrated that sertraline prevents the function of the mitochondrial VDAC1, reducing cellular ATP, increasing the ADP/ATP ratio, and activating autophagy through mTOR^80^. Our observations indicate that low doses of sertraline can reduce the phenotype of neurodegenerative disease, likely by inducing low-level mitochondrial damage. This damage may trigger PINK-1 stabilization and mitochondrial recycling.

Sertraline increases survival and neurogenesis at pharmacologically relevant concentrations in several murine models of Huntington’s^81,82^ and physiological outcomes (e.g., grip strength, coordination, locomotor activity, etc.) in rat models of Huntington’s and Parkinson’s diseases^83,84^. Given the frequent co-occurrence of depression with NDDs, it is not surprising that sertraline is often prescribed to patients suffering from both. Promisingly, some Parkinson’s patients receiving sertraline have shown improvement in their Parkinson’s symptoms^85,86^. Given these findings, it is clear that a more systematic study of the potential for sertraline for the treatment of neurodegenerative diseases is warranted.

The remaining six compounds have received considerably less characterization. In the absence of other clues, we turned toward chemical profiling approaches to attempt to predict the function of the molecules. Molinspiration, a Bayesian model that uses pharmacologically-verified training sets to predict activity against a panel of important drug targets (i.e., G-protein coupled receptors, ion channels, kinases, nuclear hormone receptors, and proteases) predicted no activity against any of these targets for any of these six compounds. It did indicate a much higher probability that sertraline would have activity against G-protein coupled receptors or ion channels.

A second cheminformatics algorithm, Prediction of Activity Spectra for Substances (PASS) that uses Bayesian analysis to predict compound function based on a training set of over 30,000 compounds was also consulted^87^. This algorithm indicated that the compounds are similar to treatments for muscular dystrophy (PS30), anti-inflammatories (PS83, PS143), complex III inhibitors (PS127, PS135), protease inhibitors (PS127), cytochrome P450 targets (PS127), and antiseborrheic treatments (PS34). With the exception of the prediction that PS127 and PS135 disrupt the function of complex III, it is difficult to unambiguously assign a molecular prediction to these functions.

PS83, formally known as [4-[*N*-[(*E*)-2-cyano-3-oxo-3-thiophen-2-ylprop-1-enyl]-S-methylsulfonimidoyl]phenyl]4-chlorobenzoate, has several similarities in treatment outcome to sertraline and triclosan. For example, all three compounds caused relatively minor mitochondrial fragmentation, but little other effect was detected here. PS103 and PS106 caused greater depolarization, while PS83 apparently did not. Searching the literature for other reports of PS83 failed to provide additional information, but the PASS characterization of PS83 as having an anti-inflammatory-like structure may provide a clue. Several anti-inflammatories have been linked with disruptions to mitochondrial function^88,89^, and the well-known cyclooxygenase-2 inhibitor celecoxib has been shown to trigger apoptosis via mitochondria-mediated pathways^90^. It is tempting to speculate that PS83 is damaging mitochondria, perhaps in some way that has not been captured here.

Two of the compounds, PS30 and PS135, seem likely to be mitochondrial uncouplers. They reduced mitochondrial membrane potential and decreased ATP content, but oxygen respiration continued unabated. Like PS83, relatively little is known about PS30 or PS135. However, an analog of PS30, known as SMTC1100, has been shown to be helpful in Duchenne muscular dystrophy^91^. This fatal, progressive disorder is characterized by wasting muscle loss due to disruption of the dystrophin protein, which leads to mitochondrial dysfunction^92,93^. This suggests that a larger portion of the scaffold may have a positive effect on mitochondrial recycling in chronic degenerative disorders. In support of this hypothesis, PASS analysis suggested that PS30 and PS135 may be useful in the treatment of neurodegenerative disease (Pa > 0.3).

The final group of three compounds, PS34, PS127, and PS143 share a number of characteristics that indicate that they may be acting the same way. They show substantial mitochondrial fragmentation, autophagolysosomal acidification, they reduced ATP and oxygen consumption, and considerably reduced average mitochondrial mass. The compounds did not, however, reduce mitochondrial membrane potential, suggesting that they disrupt degradation of PINK-1/PINK1 in a different fashion. Interestingly, they were also amongst the strongest activators of GST-4/Nrf and DAF-16/FOXO, which may indicate that they are causing other damage to the cells.

The accumulation of mitochondrial damage, and concomitant degradation of function, is associated with both aging and neurodegenerative disease. Mitophagy also appears to be inherently limited in mature neurons (reviewed in^69^), which may explain why this tissue is more sensitive to mitochondrial damage in the first place. Increasingly, it has been hypothesized and demonstrated that increasing mitophagy in these cells may promote better cellular health and aging (reviewed in^69^).

Unfortunately, a relative dearth of compounds appropriate for this purpose is currently available, and identification of new compounds requires a relatively complex screening process, like the whole-organism phenotypic approach demonstrated herein. Although the eight compounds we identified and studied have considerable promise (especially sertraline), substantial additional study is needed to further understand their effects.

## Methods

### *Caenorhabditis elegans* strains and maintenance

Worms were synchronized by hypochlorite isolation of eggs from gravid adults, followed by hatching of eggs in S Basal. 6000 synchronized L1 larvae were transferred onto 10 cm standard nematode growth medium (NGM) plates seeded with *Escherichia coli* strain OP50 as a food source^94^. After transfer, worms were grown at 20 °C for 50 h prior to experiments, or for three days for the next eggs isolation. Young adult worms were used for all assays unless otherwise noted. Strains used in this study include: N2 Bristol (wild-type), NVK90 |*pink-1*(*tm1779*); *houIs001* {*byEx655* [*Ppink-1*::PINK-1::GFP + *Pmyo-2*::mCherry]}|^9^, SJ4103 {*zcIs14* [*Pmyo-3*::GFP^mt^]}^95^, VK1241 {*vkEx1241* [*Pnhx-2*::mCherry::LGG-1 + *Pmyo-2*::GFP]}^96^, ALF89 {*Pmyo-3*::Rosella^mt^::unc-119}, TJ356 {*zIs356* [*Pdaf-16*::DAF-16a/b::GFP + *rol-6*(*su1006*)]}^55^, CL2166 {*dvIs19* [*Pgst-4*::GFP::NLS]}^97^, AY101 {*acIs101* [*Pirg-5*::GFP + *rol-6*(*su1006*)]}^98^, PE255 {*feIs5* [*Psur-5*::luciferase::GFP + *rol-6*(*su1006*)]}, GMC101 {*dvIs100* [*Punc-54*::A-beta-1–42::*unc-54* 3′-UTR + *Pmtl-2*::GFP]}^62^, GF66 {*dgEx66* [*Pvha-6*::Q82::YFP + *rol-6*(*su1006*)]}^64^, and SS104 [*glp-4*(*bn2*)]^99^.

### Bacterial strains

Bacterial strains used in this study included *E. coli* OP50, RNAi-competent OP50 (*xu363*), and RNAi-competent *E. coli* HT115 (obtained from the Ahringer RNAi library). Plasmids were isolated, purified, and sequenced prior to transformation into RNAi-competent OP50 (*xu363*). Transformed bacteria were confirmed to contain the plasmid of interest by sequencing as well.

### RNA interference protocol

RNAi-expressing bacteria were cultured and seeded onto NGM plates supplemented with 25 μg/mL carbenicillin and 1 mM IPTG. For double RNAi, bacterial cultures expressing either *vector(RNAi)* or *pink-1(RNAi)* were mixed with sterility-inducing *cdc-25.1(RNAi)* with a 1:1 ratio. For experiments with GMC101 strain, 2000 synchronized L1 larvae were plated onto 6 cm RNAi plates and grown at 20 °C for 48 h prior to use for experiments. For experiments with GF66 strain, 50 gravid hermaphrodites were transferred onto 10 cm *cdc-25.1(RNAi)* plates and grown at 20 °C for 3 days or until progenies have reached the L4 stage. Sterile progenies were then transferred to treatment plates for the next experimental step. For experiments with any other strains (SJ4103, ALF89, and N2 Bristol), 8000 synchronized L1 larvae were plated onto 10 cm RNAi plates and grown at 20 °C for 54 h prior to use for experiments.

### Statistical analysis

RStudio (version 3.6.3) was used to perform statistical analysis. Chi-square test was used to calculate the significance between qualitative variables. One-way or two-way analysis of variance (ANOVA) was performed to calculate the significance of a treatment when there were three or more groups in the experimental setting. To follow, Dunnett’s test (R package DescTools, version 0.99.34) was performed to calculate statistical significance or *p* values between each group of the statistically significant experimental results. Student’s *t*-test analysis was performed to calculate the *p* values when comparing two groups in an experimental setting. All statistical test results were indicated in graphs as follows: NS not significant, *p < 0.05, **p < 0.01, and ***p < 0.001. For each of the experiments described below, at least three biological replicates were performed.

### *Caenorhabditis elegans* library screening and compound exposure assays

Synchronized sterile young adult worms were washed from NGM plates seeded with OP50 into a conical tube and rinsed three times. Worms were then sorted into a 384-well plate (~ 25 worms/well) for the initial screen or a 96-well plate (~ 100 worms/well, half-area) for all other assays (with *C. elegans* reporter strains). For the initial high-throughput screen, each well in the 384-well plates contained 50 µM of compounds, and the exposure length was 24 h. For other compound/chemical exposure assays, S Basal supplemented with 50 µM PS compounds, 7 mM sodium selenite (Alfa Aesar), 10 µM CCCP (Sigma), 50 µM rotenone (Sigma), 50 µM juglone (Sigma), 100 µM RPW-24, or 0.1% DMSO (solvent control) was added into the wells of the 96-well plate to a final volume of 100 µL. Each treatment was performed in four wells (totaling ~ 400 worms/replicate) and in three biological replicates. Worms were imaged with Cytation5 Cell Imaging Multi-Mode Reader (BioTek Instruments) every day for three days for the initial screen or every two hours for twenty four hours for the other assays.

### Imaging and fluorescence quantification

For visualization of the worm reporter strains NVK90, TJ356, CL2166, AY101, and GF66, Cytation5 automated microscope was used. All imaging experiments were performed with identical settings within three biological replicates of each strain, with 400 animals per replicate. GFP quantifications were performed by using an image analysis pipeline of the Gen5 3.10 software and CellProfiler. For DAF-16/FOXO nuclear localization, the number of worms with punctate (indicating nuclear localization) were manually counted and divided by the total number of worms in each replicate. For visualization of the worm reporter strains SJ4103, VK1241, ALF89, and GF66, worms were immobilized by using 1 mM levamisole and then transferred onto 3% agarose pad. ~ 30 worms per replicate and at least three biological replicates were imaged using fluorescence microscope (Zeiss ApoTome.2 Imager.M2, Carl Zeiss, Germany) with a 63 × (SJ4103), 40 × (VK1241 and ALF89), or 10 × (GF66) objective magnification. For mitochondrial fragmentation in SJ4103 strain, the second muscle cell from the tail of each animal was assessed. For mCherry::LGG-1 assay, the intestine of each animal was imaged and quantified. For quantification of GFP/RFP ratio in the ALF89 strain, ImageJ was used^100^. In short, region of interests (mitochondria in the head of the worms) was determined, followed by measurement of green and red signals. Signals were normalized to the area of the region of interests and ratio of green/red was calculated for each worm.

### Fluorescent-dye staining and quantification

Approximately 400 N2 worms (in four wells, each contain ~ 100 worms) were treated with PS compounds or corresponding controls for 15 h in 96-well plate. At 14 h of incubation, fluorescent dye with a final concentration of 10 µM for LysoTracker Red, 4.375 µM for MitoTracker Green, 4.375 µM for MitoTracker Red, or 3 µM for DHE, were added. For background subtraction, S Basal without any dye (with corresponding concentration of DMSO solvent (final concentration of 1% for LysoTracker Red, MitoTracker Green, and MitoTracker Red, and 0.04% for DHE)) was added. Worms were washed three times to remove any remaining compounds or dye before fluorescence measurement was taken via flow vermimetry (COPAS Biosort, Union Biometrica). Fluorescence values were normalized to DMSO control (set at 100%).

### ATP production measurement

A worm strain carrying firefly luciferase gene followed by GFP (PE255) was used for ATP production measurement. Worms were treated with compounds as described above (~ 100 worms/well, four wells per condition per biological replicate, and three biological replicates were performed). ATP measurement was carried out according to the published protocol^101^. Essentially, at 18 h of incubation, worms were washed three times to remove any remaining compounds. Luminescence buffer was then added, incubated for 3 min, and fluorescence (485/20 excitation and 528/20 emission) and luminescence were measured with Cytation5 (BioTek Instruments).

### Oxygen consumption rate measurement

3000 N2 worms were sorted into each well of a 6-well plate. PS compounds, vehicle control DMSO, or positive control rotenone, and *E. coli* OP50 (final OD_600_: 0.05) were then added into each well to a final concentration of 50 µM. Two wells for each compound were used, totaling to 6000 worms per condition. Upon 8 h of incubation, worms were collected and transferred into a 15 mL conical. Worms were washed three times to remove residual compounds. Oxygen consumption was measured by using a biological oxygen monitor (YSI 5300) and a Clark-type oxygen electrode (YSI 5301) (Yellow Springs Instrument) at 20 °C as previously described^49^. Oxygen consumption was recorded continuously for ten minutes.

### β-Amyloid-induced paralysis and scoring

Synchronous L1 population of *C. elegans* strain GMC101^102^ was reared on *E. coli* OP50 at 20 °C for 48 h to reach the L4 stage. 60 worms were then transferred onto each 35 mm NGM plates containing 250 µM 5-fluoro-2’-deoxyuridine (FUDR) and PS compounds (see Fig. S2 for final concentrations) or corresponding DMSO control. Worms were then incubated at 25 °C to induce paralysis and scored every day for five days. Paralysis was indicated by inability to complete a full sigmoidal body movement spontaneously or following stimulation with a pick. Paralyzed worms were counted and removed from the plate.

### Polyglutamine protein aggregation scoring

50 gravid hermaphrodites of *C. elegans* strain GF66 expressing polyglutamine (*Pvha-6*::Q82::YFP)^64^ were reared on *E. coli* OP50 expressing *cdc-25.1(RNAi)* and grown at 20 °C for 3 days or until progenies have reached the L4 stage. L4 worms were then transferred onto treatment plates as for the beta-amyloid-induced paralysis assay, but kept at 20 °C. After 36 h, worms were transferred into a 96-well plate, washed three times, immobilized with 1 mM levamisole, and imaged with Cytation 5 automated microscope with a 4 × objective magnification. The number of aggregates were counted manually for each of the worms.

### *Caenorhabditis elegans* compound toxicity assay

25 synchronized SS104 (*glp-4*) young adult worms were sorted into 384-well plate. Compounds (final concentration: 100 µM, 50 µM, 25 µM, and 12.5 µM) were mixed with *E. coli* OP50 as food source (final OD_600_: 0.05), and then added into each well (20 wells per condition per biological replicate and three biological replicates were performed). Plates were incubated at 25 °C for 72 h. Plates were washed three times and worms were stained with SYTOX™ Orange nucleic acid dye to stain dead worms. After 14 h of incubation with SYTOX™, plates were washed and imaged with Cytation5 automated microscope. CellProfiler software was used to quantify worms’ death.

### Mammalian cell culture and compound toxicity assay

Human fetal glial cells (SVGp12) and prostate epithelial cells (RWPE-1) were purchased from ATCC (Manassas, VA, USA). SVGp12 cells were cultured in minimum essential Eagle medium (ThermoFisher) containing 10% FBS (fetal bovine serum; Corning, Manassas, VA, USA) and RWPE-1 cells were cultured in Defined Keratinocyte SFM (ThermoFisher) with growth supplement at 37 °C in a humidified 5% CO_2_ atmosphere. The solution of penicillin–streptomycin (Gibco, Gaithersburg, MD, USA) was used at 1% final concentration.

Fluorescent cell labeling with Hoechst 33342 (ThermoFisher) and propidium iodide (ThermoFisher) with subsequent automated cell counting was used as cytotoxicity assay as previously described^68^. Cytation5 Cell Imaging Multi-Mode Reader with DAPI and Texas Red filter sets (BioTek, Winooski, VT, USA) and Gen5 software v. 3.00 were used for imaging and cell counting pipeline.

For each cytotoxicity experiment, cells were seeded at a density of 10^3^ cells/well in 96-well plates and cultured for 24 h prior to the drug treatment. Cells were then treated with PS compounds or DMSO (solvent control) at specified concentrations (see Fig. 7) for 72 h in 100 µL of complete media. All viability rates were normalized to the corresponding solvent-control wells. The DMSO concentrations in the incubation mixtures or solvent-control wells never exceeded 0.5% (v/v).

## Supplementary Information

Supplementary Information.
