# Supplementary material for: Mitochondria-affecting small molecules ameliorate proteostasis defects associated with neurodegenerative diseases
Source: Sci Rep. 2021 Sep 6;11:17733. doi: 10.1038/s41598-021-97148-z (PMC8421394; doi:10.1038/s41598-021-97148-z)
Supplement: Supplementary file 1 — Supplementary Information. [file 41598_2021_97148_MOESM1_ESM.pdf]

PS30

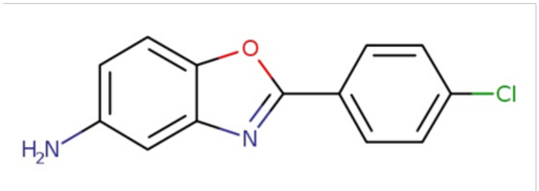

PS34

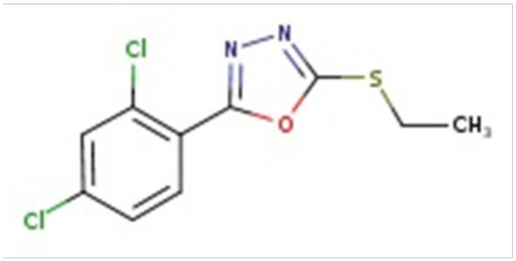

PS83

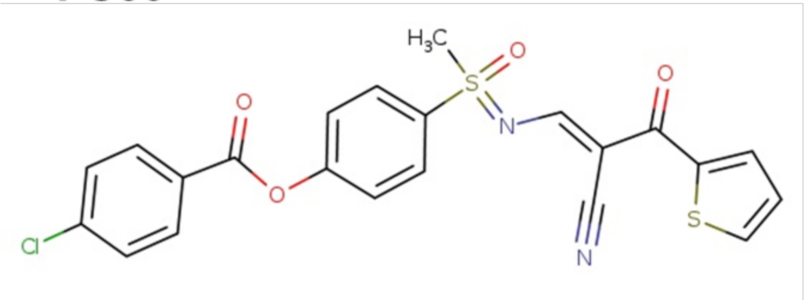

PS103

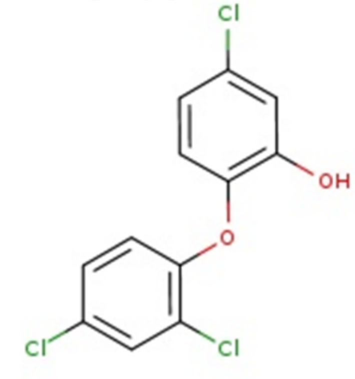

PS106

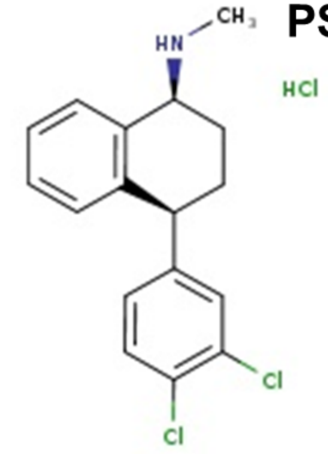

PS127

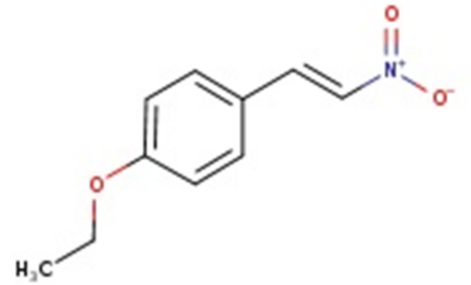

PS135

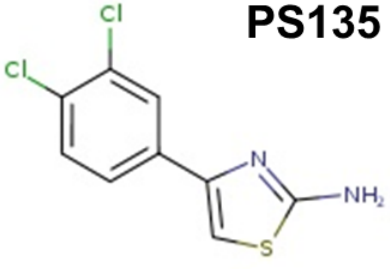

PS143

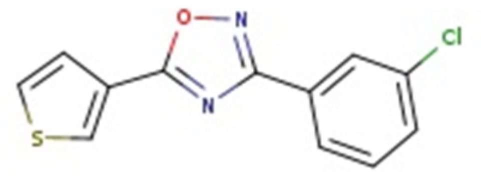

Figure S1. Chemical structures of the eight PS compounds.

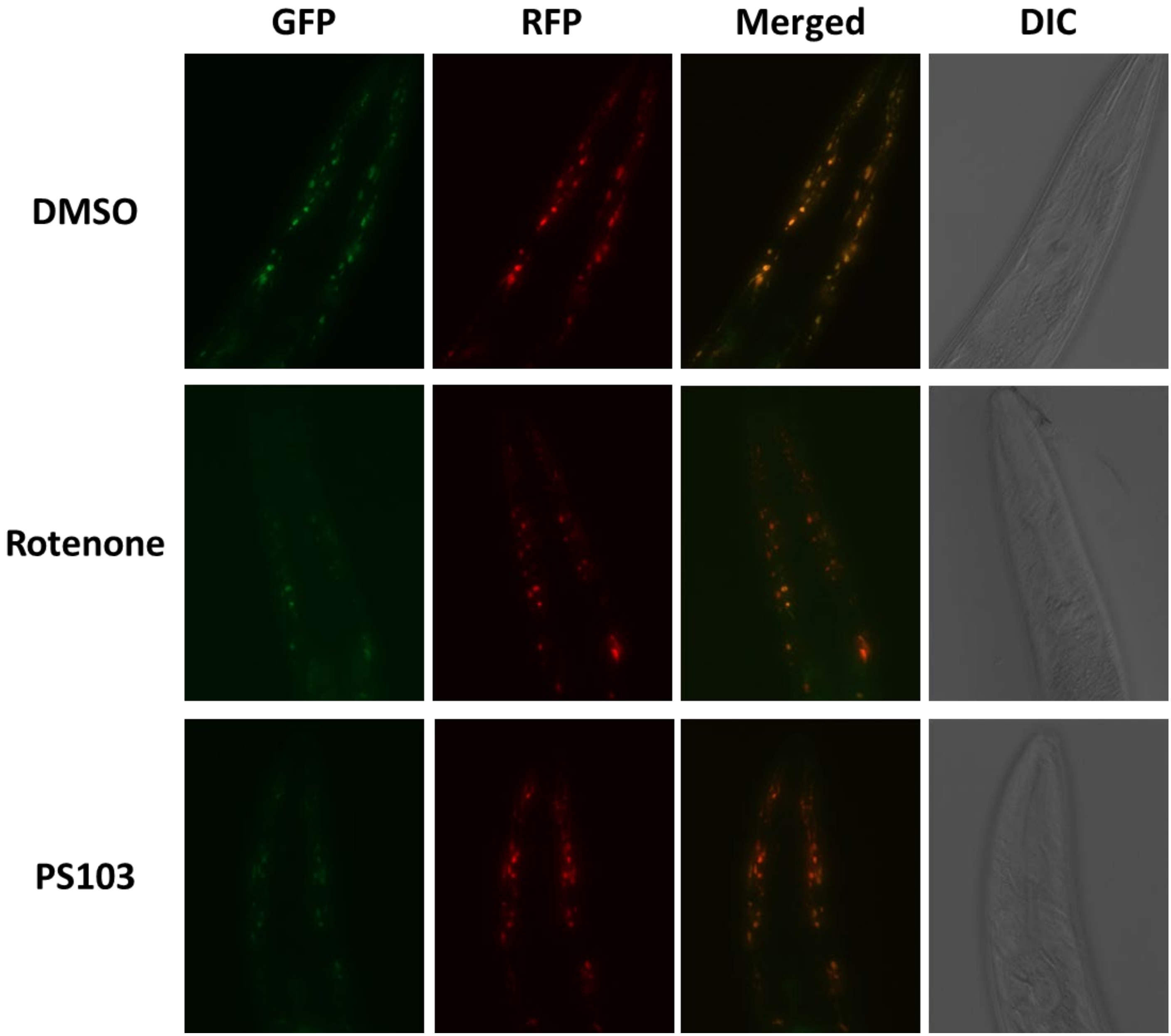

Figure S2. PS compounds induced mitophagy as observed by utilizing the Rosella^{mt} reporter. Fluorescent (GFP, RFP, and merged) and DIC images of *C. elegans* strains expressing mitochondria-localized Rosella biosensor upon treatment with DMSO, 50 μM rotenone, or 50 μM PS103.

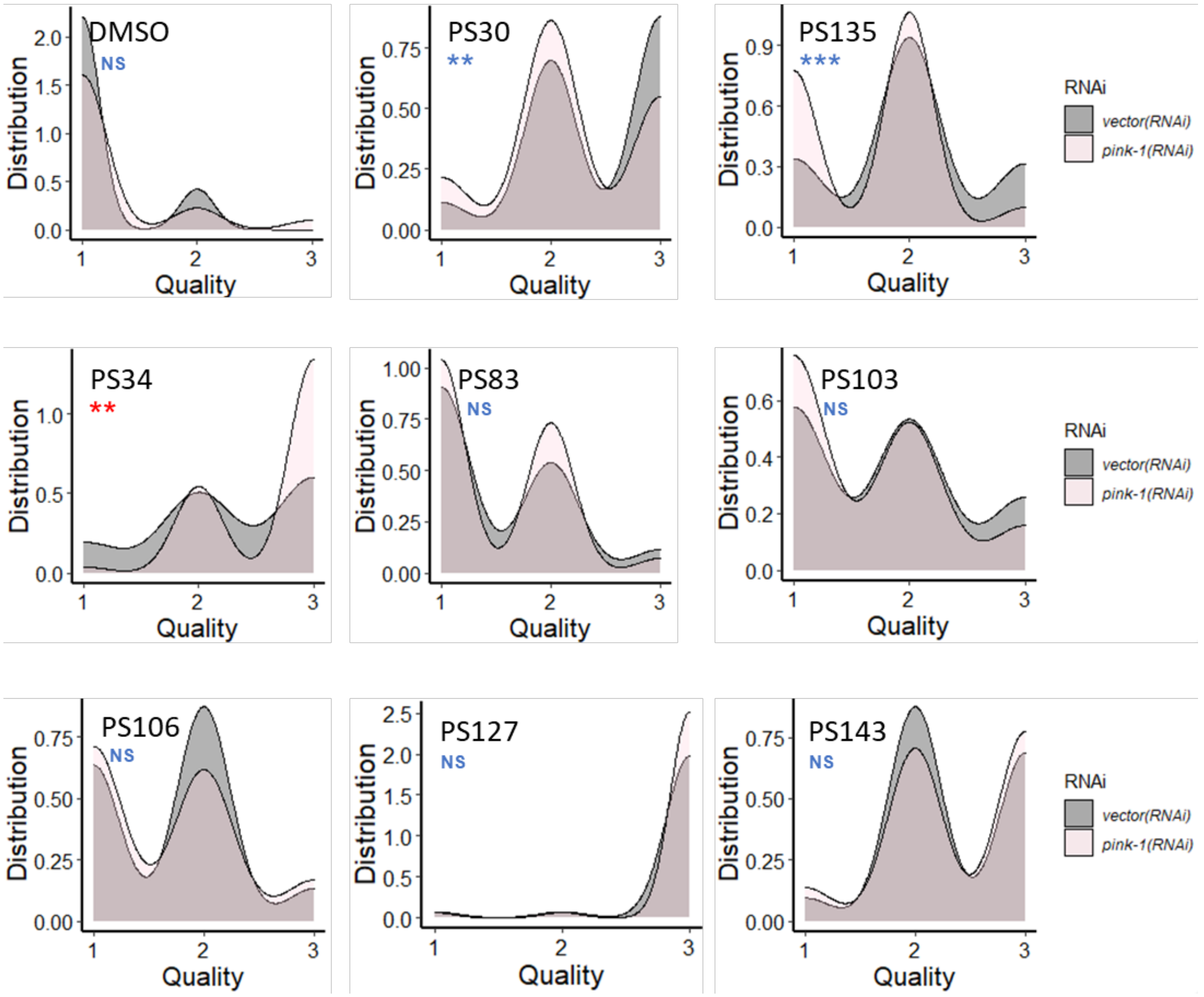

Figure S3. Two PS compounds, PS30 and PS135, were partially dependent on PINK-1/PINK1 in inducing mitochondrial fragmentation. Distribution plot of mitochondrial quality upon 18 h of exposure to PS compounds or DMSO control in *C. elegans* carrying *Pmyo-3*::GFP^{mt} reared on empty vector or *pink-1(RNAi)*. Chi-square statistic tests were performed, and *p*-values were indicated on graphs. Three biological replicates of ~20 worms/replicate were analyzed.

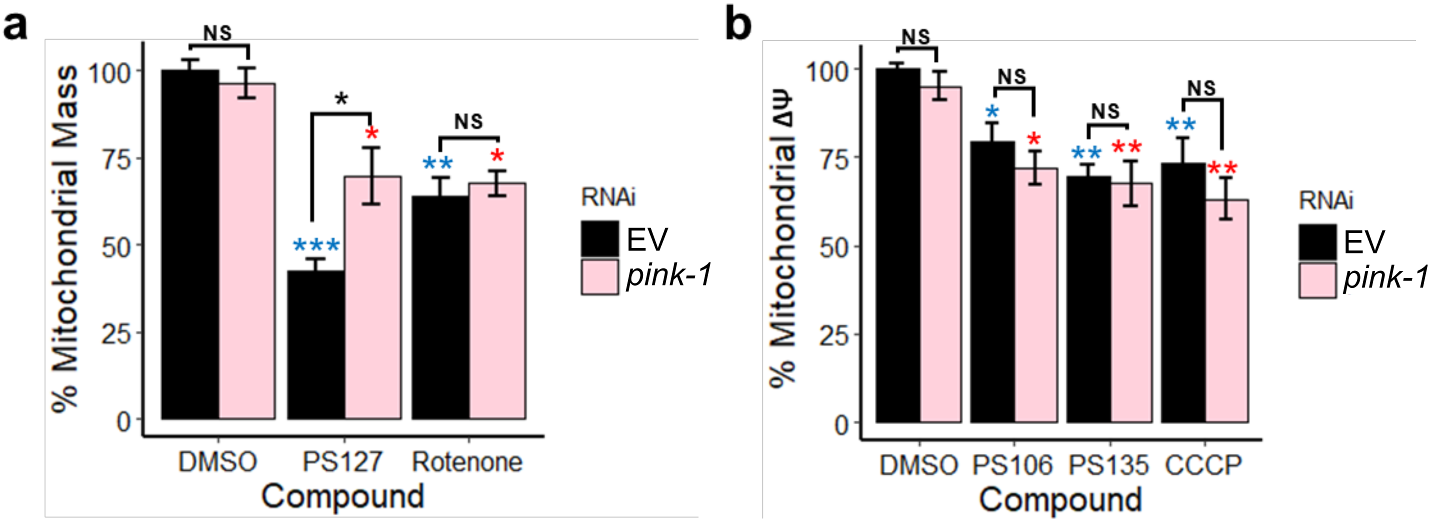

Figure S4. PS compounds' act via multiple mechanisms. (a, b) Fluorescent quantification of **(a)** MitoTracker Green (mitochondrial mass) or **(b)** MitoTracker Red (mitochondrial membrane potential) of wild-type worms reared on vector (EV) or *pink-1(RNAi)* upon 15 h of treatment with **(a)** 50 μM PS127 or 50 μM rotenone or **(b)** 50 μM PS106, 50 μM PS135, or 10 μM CCCP. At least three biological replicates with ~400 worms/replicate were analyzed. *p* values were determined from two-way ANOVA, followed by Dunnett's test. All fold changes were normalized to EV-DMSO control (at 100%). NS not significant, **p* < 0.05, ** *p* < 0.01, *** *p* < 0.001. Blue stars indicate significance of change compared to EV-DMSO, red stars indicate significance of change compared to *pink-1(RNAi)*-DMSO, and black stars indicate significance of change between EV and *pink-1(RNAi)* for each compound.

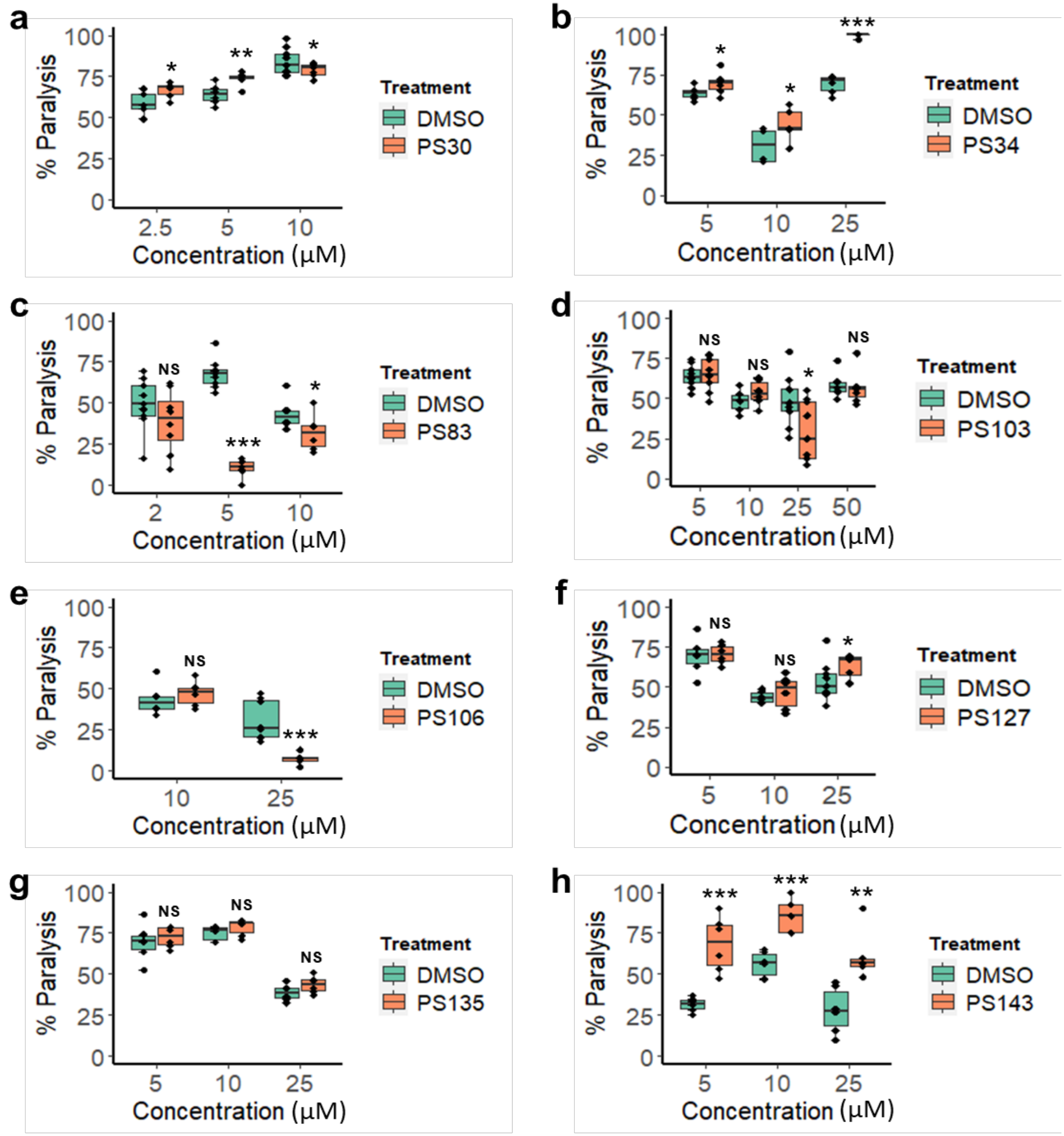

Figure S5. Three PS compounds reduced the rate of paralysis in *C. elegans* Alzheimer's model. Percent paralysis of *C. elegans* GMC101 that expresses full-length human beta-amyloid upon treatment with **(a-h)** DMSO control compared to **(a)** PS30, **(b)** PS34, **(c)** PS83, **(d)** PS103, **(e)** PS106, **(f)** PS127, **(g)** PS135, and **(h)** PS143. Concentrations of PS compounds were indicated on the graphs. At least three biological replicates with ~180 worms/replicate were analyzed. p values were determined from Student's t-test. NS not significant, *$p < 0.05$, ** $p < 0.01$, *** $p < 0.001$.

Table S1. Chemical information of the eight PS compounds.

Compound	Library Source	MW (Da)	LogP	Polar area	Donor	Acceptor	Bonds
**PS30**	ChemBridge	244.67	3.4	52.0	1	3	1
**PS34**	ChemBridge	275.15	3.9	64.2	0	4	3
**PS83**	Maybridge	471.00	5.5	133.0	0	7	7
**PS103**	NIHCC	289.50	5.0	29.5	1	2	2
**PS106**	UT_Kinase	342.70	5.1	12.0	2	1	2
**PS127**	ChemBridge	193.20	2.6	55.0	0	3	3
**PS135**	ChemBridge	245.13	3.5	67.2	1	3	1
**PS143**	Maybridge	262.72	3.8	67.2	0	4	2

Table S2. Tanimoto coefficient of the eight PS compounds as compared to each other.

	PS30	PS34	PS83	PS103	PS106	PS127	PS135	PS143
**PS30**	1							
**PS34**	0.291	1						
**PS83**	0.134	0.158	1					
**PS103**	0.104	0.139	0.144	1				
**PS106**	0.192	0.167	0.157	0.135	1			
**PS127**	0.119	0.126	0.183	0.218	0.110	1		
**PS135**	0.218	0.241	0.175	0.128	0.195	0.115	1	
**PS143**	0.192	0.184	0.191	0.096	0.178	0.102	0.267	1
